# Criterion validity and reliability of the International Physical Activity Questionnaire – Hungarian short form against the RM42 accelerometer

**DOI:** 10.1186/s12889-021-10372-0

**Published:** 2021-04-23

**Authors:** Pongrác Ács, Réka Veress, Paulo Rocha, Tamás Dóczi, Bence László Raposa, Petra Baumann, Sergej Ostojic, Viktória Pérmusz, Alexandra Makai

**Affiliations:** 1 Faculty of Health Sciences, University of Pécs, Vörösmarty u. 4, Pécs, H-7621 Hungary; 2 Hungarian Leisure Sport Association, Istvánmezei út 1-3, Budapest, H-1146 Hungary; 3 Instituto Português do Desporto e Juventud, 1990-100 Lisbon, Portugal; 4 Faculdade de Motricidade Humana, Universidade de Lisboa, 1649-004 Lisbon, Portugal; 5 University of Physical Education, Budapest, Hungary

**Keywords:** EUPASMOS, Physical activity, Monitoring, IPAQ-SF, RM42, Accelerometer, Validation

## Abstract

**Background:**

Physical inactivity is a global phenomenon in European welfare countries. Proper monitoring is essential to measure the physical activity level of the population.

**Methods:**

In the Hungarian cohort of the European Physical Activity and Sport Monitoring System (EUPASMOS) project, our participants (*N* = 598) completed sociodemographic questions and the International Physical Activity Questionnaire – short form (IPAQ-SF) survey. The validity and reliability of the subjective measurement tool were examined, IPAQ-SF outcomes were contrasted against triaxial RM42 accelerometer wore for 7 consecutive days.

**Results:**

The IPAQ-SF showed moderate internal consistency (Cronbach Alpha = 0.647). The concurrent validity of the IPAQ-SF to triaxial accelerometer indicated a significant weak-to-moderate correlation (R = 0.111–0.338, *p* = 0.042; *p* < 0.001). The test-retest reliability showed a significant correlation between two measurements (R = 0.788–0.981, p < 0.001).

**Conclusion:**

The Hungarian version of the IPAQ-SF had excellent test-retest reliability, but low-to-fair concurrent validity for moderate and vigorous physical activity, walking and sitting time, as compared to the objective criterion measure among Hungarian adults.

## Background

Physical inactivity is a global phenomenon in the European countries. Sedentary lifestyle causes 5.3 million deaths worldwide each year [1]. In Hungary only one third of the working age population meets the WHO standards on Global *recommendations* on *physical activity (PA) for health* [2, 3]. The economic burden of the inactivity is high [4]. The social and economic effect of inactivity urges the need for adequate monitoring of physical activity (PA) and the elaboration of a national guidelines of PA for healthy adults [5]. Population level PA monitoring may support public health benefits. Testing the validity of physical activity questionnaires (PAQs) is crucial to inform researchers when presenting the relationship between self-reported PA and health outcomes [6].

Furthermore, in their study, Sanda et al. (2017) highlighted that the general population rarely met the developed national or international guidelines. Moreover, not only is the scientific output of the monitoring essential, but society should also be informed of the results of a PA monitoring and directed to a physically active lifestyle.

The IPAQ-SF is one of the most frequently used questionnaires for examining the self-reported PA of the general adult population [7]. The popularity of the self-reported method is also continuous in clinical practice, as it is a short and cost-effective way for measuring PA levels in patients [8, 9].

The psychometric properties of the Hungarian version of the questionnaire are not known. Our study aims to test the reliability and validity of the Hungarian version of the IPAQ-SF questionnaires in a population-based sample of healthy adults in Hungary, to investigate the reliability of 7-days test-retest, and to compare the questionnaire data with an objective measurement device, the RM42 triaxial accelerometer.

## Methods

### Study design

In our validation study we used the data recorded in the framework of the EUPASMOS project [10]. The EUPASMOS is an international cohort study involving 18 European countries, including Bulgaria, Cyprus, Denmark, England, Finland, France, Hungary, Italy, Latvia, Macedonia, Malta, the Netherlands, Poland, Portugal, Romania, Slovenia, Spain and Sweden. Our data analysis is based on the Hungarian data collection.

### Participants

We invited 800 adult participants from Budapest and the South Transdanubia region to participate in our study between October 2018 and October 2019.

The sample was recruited by advertising and distribution of written study information by the University of Physical Education and University of Pécs. Interested individuals were contacted by an interviewer in person. All participants received written information about the purpose of the study and were instructed in the proper use of the RM42 accelerometer and completed a consent form. Based on the EUPASMOS project guidelines we sought quota sampling regarding gender and age, in four age groups in equal proportions, however, there were difficulties in achieving male respondents especially above the age of 65.

A total of 626 adults participated in the study, who received a collection of the questionnaires and the RM42 accelerometers. The final convenience sample size with completed questionnaires and appropriate accelerometer data, included 598 participants. The Hungarian data set contained retest data from 80 persons (14 days after the first round) from a heterogenous sample with different socio-economic status. The first 80 participant with completed questionnaires, appropriate accelerometer data and with interest on the second round were sampled consecutively in the retest subsample.

### Collection of the data

The demographic questionnaire contained the main information about the participants socio-economic status based on the European Health Interview Survey (EHIS) [11]. Age, gender, education, marital status, place of residence was studied. Furthermore, we examined the general health status using a 5-point Likert scale [11]. The height and the weight of the participants were measured in light clothing without shoes. Body mass index (BMI) was calculated (kg/m2) and defined as normal and underweight (< 25 kg/m2), overweight (25 to 29.9 kg/m2) and obese (≥30 kg/m2) [12].

### Assessing the level of physical activity

The level of PA was measured by IPAQ-SF questionnaire. The participants reported information on the average amount of vigorous and moderate intensity PA, walking activities, and time spent sitting. The seven questions provided information about the intensity of the PA and the sedentary time according to the frequency and duration of the activities. The reported amounts were registered in minutes per week, except sitting time which was reported in minutes per day.

7-day IPAQ-SF is a standardized measurement tool of PA behaviour in various populations [13, 14].

### Accelerometry data

A triaxial accelerometer (UKK RM42, Institute for Health Promotion Research, Tampere.

Finland) was used along with the new method to process the raw data [15]. UKK RM42 is a lightweight triaxial accelerometer that was attached to an elastic belt and worn on the right side of the hip resting on the iliac crest during waking hours (excluding the time spent in the sauna, bath, shower, or in other water activities). The accelerometers were worn on the non-dominant hand with a wristband during the sleep time. The angle for postural estimation (APE) analysis was performed to distinguish between stationary sitting and standing.

Partner country data were uploaded to a country specific UKK cloud. The UKK processed the algorithms and analysed the data. After this process, the UKK reloaded the analysed data back into to the country-specific cloud.

### Statistical analyses

Continuous descriptive data were presented as mean (SD) and median (interquartile range), while data for qualitative characteristics were expressed as percent values. The normality of the data was tested by Kolmogorov-Smirnov tests. Gender differences were tested using Mann-Whitney U tests. The relationship between the continuous variables was measured by Spearman’s rank correlation. The validity of the questionnaire was measured by Cronbach alpha coefficient, where > 0.5 and > 0.7 were considered acceptable and good, respectively. Spearman’s rank ordered correlation coefficients (ρ) were calculated to assess concurrent (inter-method) validity and the following reference values were used for interpretation: 0–0.20 weak correlation, 0.21–0.40 fair, 0.41–0.60 moderate/acceptable, ≥0.6 strong [16]. We measured the differences between the IPAQ-SF 1 test and IPAQ-SF 2 retest results using the Wilcoxon rank test. The strength of agreement between the subjective and objective methods was determined using the Bland-Altman plot with the limits of agreement technique, which provided the mean bias and the 95% limit of agreement (±2 standard deviation (SD) of the difference), presented as the level of agreement between the IPAQ-SF and the RM42. The relationship between the differences and mean of the measurement tools were calculated by using a linear regression analysis. The significance level was set at *p* < 0.05. We used IBM SPSS 24.0 software to analyse the results.

## Results

### Baseline characteristics of study participants

Socio-demographic and behavioural data of the study participants are shown in Table 1. The mean age of the men and women was 41.06 (16.63) and 45.07 (16.23) years, respectively. The average BMI was 25.24 (4.84). In terms of the place of residency, 68.92% lived in towns or cities. Majority of the participants completed secondary or higher education (99.31%). We found significant gender differences in the study population by age, marital status and general health. Gender differences represented the Hungarian age groups.

**Table 1:** Baseline characteristics of study participants

		Total		Male		Female		Gender differences (p)
		**N**	**%**	**N**	**%**	**N**	**%**	
**Age groups**	**18–34**	185	30.94	91	35.69	94	27.41	0.001
	**35–49**	169	28.26	79	30.98	90	26.24	
	**50–64**	140	23.41	59	23.14	81	23.62	
	**65+**	104	17.39	26	10.20	78	22.74	
**Marital status**	**single**	135	22.88	60	23.81	75	22.19	0.006
	**married or cohabitated**	358	60.68	164	65.08	194	57.40	
	**widow**	32	5.42	5	1.98	27	7.99	
	**divorced**	65	11.02	23	9.13	42	12.43	
**Self-reported general health**	**very good**	92	15.46	54	21.34	38	11.11	0.001
	**good**	348	58.49	148	58.50	200	58.48	
	**fair**	136	22.86	45	17.79	91	26.61	
	**bad**	15	2.52	6	2.37	9	2.63	
	**very bad**	4	0.67	0	0.00	4	1.17	
**Place of residence**	**village**	184	31.08	77	30.43	107	31.56	0.769
	**town or city**	408	68.92	176	69.57	232	68.44	
**Education**	**primary**	4	0.69	3	1.21	1	0.30	0.370
	**secondary**	338	58.18	140	56.68	198	59.28	
	**high**	239	41.14	104	42.11	135	40.42	

Reported grand total may not be equal to the sum of the subtotals due to missing data

**Fig. 1:**
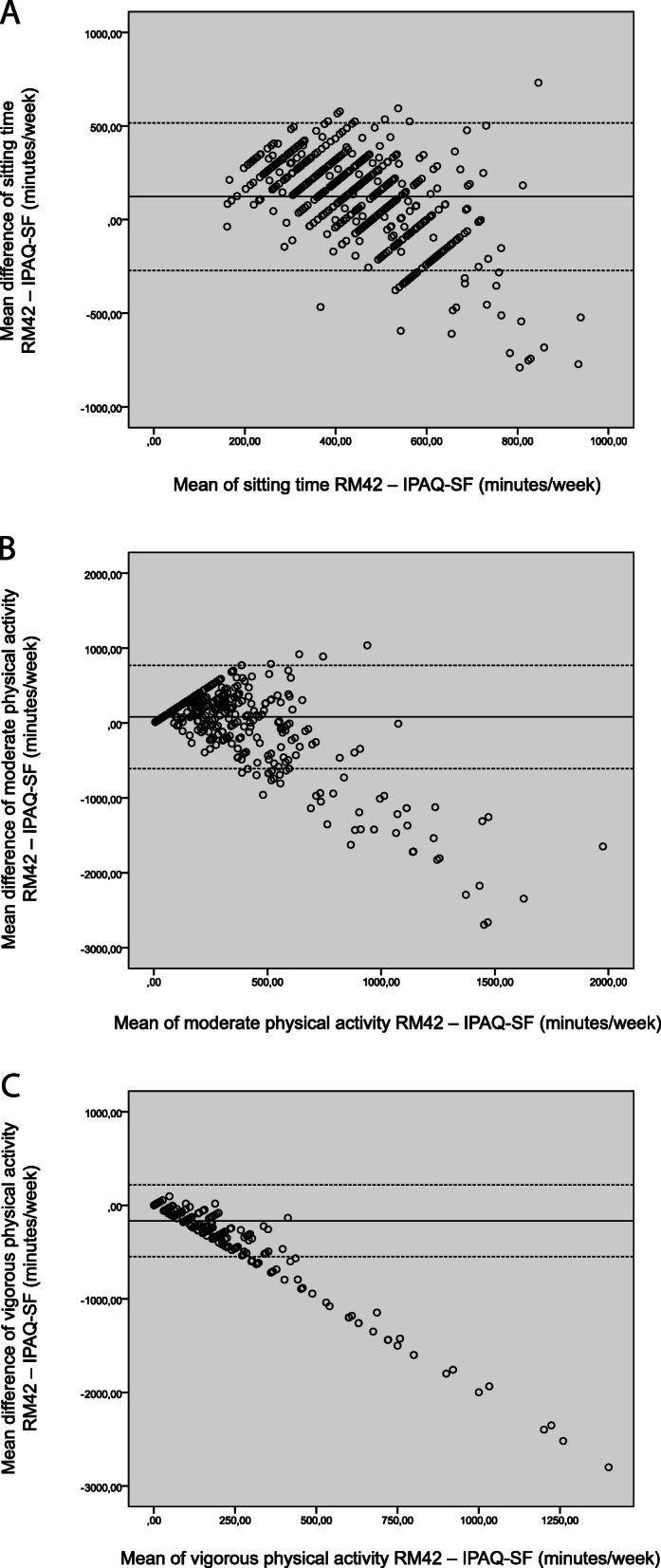
Bland Altman plots of the IPAQ-SF and RM42 accelerometer according to vigorous-, moderate activities (min / week) and sitting time (min / day). The metric for both x- and y-axes in each graph (a-c) are the z-scores for mean of the RM42 accelerometer and IPAQ-SF scores and the difference (difference of the accelerometer questionnaire scores) between scores, _______ = observed average agreement; − - - - - =95% limit of agreement; y = 0 line of perfect average agreement

Table 2 shows the main physical activity patterns of our sample measured by IPAQ-SF and RM42 accelerometer. We found significant gender differences in terms of standing, moderate, vigorous and sitting time and number of steps per day as measured by the accelerometer (*p* < 0.05). The IPAQ-SF showed significant gender differences only in amounts of vigorous intensity activities (*p* < 0.001). Male participants were more active than women, however male participants spent more time sitting.

**Table 2:** Main physical activity patterns of the sample measured with RM42 accelerometer and IPAQ-SF

	Total					Male					Female					p
	Mean	Median	SD	Percentiles		Mean	Median	SD	Percentiles		Mean	Median	SD	Percentiles		
				25	75				25	75				25	75	
**RM42 stand – min / week**	736.16	681.28	348.61	495.51	944.48	663.70	605.85	306.00	452.32	878.62	790.03	742.35	368.48	548.92	995.75	< 0.001
**RM42 - light PA – min / week**	1608.54	1548.81	552.60	1238.80	1918.58	1612.21	1534.40	561.57	1239.82	1931.88	1605.81	1554.35	546.64	1238.53	1910.65	0.848
**RM42 moderate PA min / week**	365.69	332.15	200.73	218.55	485.68	403.26	387.80	213.50	244.30	544.25	337.76	305.43	186.13	213.73	442.87	< 0.001
**RM42 vigorous PA min / week**	22.17	2.57	54.39	0.00	20.56	27.13	4.14	65.74	0.09	29.63	18.47	1.75	43.79	0.00	14.35	0.017
**RM42 Sitting time min / day**	510.99	505.31	116.58	433.41	579.86	533.55	522.73	122.81	459.37	614.12	494.22	494.28	108.92	426.52	557.10	< 0.001
**RM42 daily steps**	7259.22	6977.86	2983.85	5312.11	8856.79	7564.21	7360.14	3190.20	5655.86	9512.57	7035.69	6798.00	2806.88	5180.14	8505.36	0.013
**IPAQ-SF test vigorous min / week**	279.00	180.00	409.84	0.00	360.00	390.03	240.00	492.66	60.00	480.00	192.36	97.50	305.20	0.00	270.00	< 0.001
**IPAQ-SF test moderate min / week**	414.01	196.50	557.73	60.00	544.75	491.27	240.00	637.55	60.00	600.00	344.57	180.00	465.69	0.00	420.00	0.056
**IPAQ-SF test walking min / week**	640.44	420.00	671.53	240.00	840.00	636.58	420.00	657.29	240.00	840.00	643.72	420.00	685.86	270.00	840.00	0.798
**IPAQ-SF test sitting time min / day**	403.89	360.00	220.72	240.00	540.00	404.12	360.00	218.67	240.00	540.00	403.73	360.00	222.52	240.00	517.50	0.912

### Validity and reliability of the Hungarian IPAQ-SF questionnaire

Concurrent validity correlation coefficients of the IPAQ-SF and RM42 are shown in Table 3. Fair (nearly moderate) correlation was found between the vigorous PA measured with the RM42 and the IPAQ-SF questionnaire (R = 0.282; *p* < 0.001). Weak correlation between the IPAQ-SF and RM42 was found in case of moderate PA, but moderate PA measured by IPAQ-SF was significantly correlated with accelerometer-measured light activities. The IPAQ-SF walking time was significantly correlated with light, moderate PA (R = 0.277, *p* < 0.001) and with accelerometer step values (R = 0.338; *p* < 0.001). The time spent sitting in an average day measured by IPAQ-SF proved to be fair but there was a significant correlation with RM42 sitting time (R = 0.255; *p* < 0.001) (Table 3).

**Table 3:** Concurrent validity of the IPAQ-SF compared to RM42 accelerometer based on Spearman’s rank correlation analyses

		RM42 stand - min / week	RM42 - light PA - min / week	RM42 moderate PA min / week	RM42 vigorous PA min / week	RM42 sitting time min / day	RM42 daily steps number
**IPAQ-SF test vigorous min / week**	R	.077	.186^**^	.249^**^	.282^**^	−.141^**^	.290^**^
	p	.094	<.001	<.001	**<.001**	.002	<.001
**IPAQ-SF test moderate min / week**	R	.052	.206^**^	.111^*^	−.016	−.184^**^	.128^*^
	p	.338	**<.001**	**.042**	.774	.001	.019
**IPAQ-SF test walking min / week**	R	−.061	.157^*^	.277^**^	.023	−.065	.338^**^
	p	.344	**.014**	**<.001**	.724	.310	**<.001**
**IPAQ-SF test sitting time min / day**	R	−.109^**^	−.281^**^	−.081	−.007	.255^**^	−.134^**^
	p	.009	<.001	.054	.875	**<.001**	.001

Relevant findings were highlighted in bold

### Differences between the accelerometer and the questionnaire scores using bland-Altman plots

The Bland-Altman plots were based on One-Sample T-tests where we found that the differences between accelerometer and questionnaire scores were significantly higher than 0 in all scores studied, such as vigorous-, moderate activities and time spent sitting (*p* < 0.001).

The average difference according to the vigorous activities was − 166.21 min / week. Participants in subjective measurement overreported the time spent with vigorous intensity activities by more than 2 h. While the difference between the accelerometer and IPAQ-SF showed that the time spent with moderate activities was underreported by an average of 82.97 min / week, according to the self-reported method. Finally, the time spent sitting was also underreported by the questionnaire data against the RM42 accelerometer scores, because the average difference was 122.91 min /day, the participants’ sitting time was more than 2 h higher than reported. The results of the Bland-Altman plots were completed with linear regression analyses, where we found a significant correlation between the differences and the mean of the two measurements in each examined score, higher mean scores were associated with higher measurement difference scores (vigorous activities: R^2^ = 0.965, F = 12,733.434, *p* < 0.001; moderate activities: R^2^ = 0.551, F = 412.607, *p* < 0.001; sitting time: R^2^ = 0.329, F = 280.449, *p* < 0.001) Fig. 1.

### Test – retest validity of the IPAQ-SF questionnaire

According to Wilcoxon signed rank tests, here were no significant differences between IPAQ-SF 1 and IPAQ-SF 2 in PA min / week in terms of moderate activities (*p* = 0.130) and walking time (*p* = 0.068), although the vigorous PA time (*p* = 0.017) and sitting time (*p* = 0.001) was significantly different. However, strong correlation was found between the two measurements in vigorous, moderate, walking and sitting times (R = 0.788–0.981, *p* < 0.001). The test-retest results are shown is Table 4.

**Table 4:** Test retest reliability of the Hungarian IPAQ – SF questionnaire

IPAQ-SF Test-retest reliability		IPAQ-SF retest vigorous min / week	IPAQ-SF retest moderate min / week	IPAQ-SF retest walking min / week	IPAQ-SF retest sitting time min / day
**IPAQ-SF test vigorous min / week**	R	0.908			
	p	**p < 0.001**			
**IPAQ-SF test moderate min / week**	R		0.981		
	p		**p < 0.001**		
**IPAQ-SF test walking min / week**	R			0.788	
	p			**p < 0.001**	
**IPAQ-SF test sitting time min / day**	R				0.826
	p				**p < 0.001**

## Discussion

Our study tested the reliability and validity of the IPAQ-SF and RM42 accelerometer for use in various cohort studies in the fields of health, sports and social sciences. The Hungarian version of the GPAQ and IPAQ-LF are already valid measurement tools in Hungarian language as well [5, 17]. But we considered it important to examine the reliability and validity of the IPAQ-SF questionnaire as it is a fairly popular subjective method for measuring physical activity in such research, where the main focuses do not allow the use a longer, multi-domain questionnaire. Although, the IPAQ-SF questionnaire does not include different domains of physical activity and only collects data based on the intensity of PA, using only 7 questions, it shows relevant information.

In our study the comparison of the level of total physical activity showed a fair-to-moderate correlation between the scores of the questionnaire and the accelerometer. Nevertheless, we found significant correlation between self-reported and accelerometery-based physical activity.

The question in IPAQ regarding sitting showed weak correlation and a narrow range in mean accelerometer sedentary time (R = 0.55, *p* < 0.001). The different intensity scores of the questionnaire showed significant association with accelerometer daily number of steps.

IPAQ-SF moderate score showed stronger correlation with accelerometer-based light activity (R = 0.206, *p* < 0.001) than with accelerometer-based moderate activities.

(R = 0.111; 0.042). Furthermore, the accelerometer results showed a difference of more than two hours in vigorous activities as well as and sitting time also, but the average difference between the moderate accelerometer scores and questionnaire scores was also more than 1 h.

The IPAQ questionnaire has been also discussed most recently in relation to lockdown and social distancing caused by COVID-19. Castañeda-Babarro and co-authors studied self-reported PA, measured with IPAQ-SF, which was significantly reduced in a Spanish adult sample during confinement. However, VPA and walking time decreased (16.8%, *p* < 0.001 and 58.2%, *p* < 0.001), whereas ST increased (23.8%, p < 0.001). The ratio of respondents who met the 75 min/week of VPA recommendation decreased (10.7%, p < 0.001) while the proportion of those who achieved the 150 min/week of MPA barely changed (1.4%). Women reduced iVPA time more than men (9% vs 21%) and even increased MPA time (11%, *p* < 0.05) and reported lower increase in ST (35% vs 25.3%) [18].

Romero-Blanco et al. also studied PA and sitting time as main dependent variables, during the coronavirus pandemic and the resulting lockdown. IPAQ-SF was used for the measurement and various consequences were experienced among university students, according to the weekly PA (MD: -159.87; CI: − 100.44, − 219.31). Weekly ST also increased (MD: -106.76; CI: − 71.85, − 141.67) [18].

The questionnaire can also be used reliably in special populations or even in special stages of life or under medical conditions. Pans et al. examined this issue within a special population, among people with disabilities in a university setting to explore the relationships between screen time, degree of disability, body mass index (BMI), physical activity and sociodemographic variables. 5.45 h/day of total screen time was reported which was slightly higher for women than for men and lower for those with high degrees of disability. Contradictory results were found for genders. The group of men with the highest BMI had the highest screen time and the lowest PA while women with low BMI had the least ST and the lowest PA [19].

Pregnancy as a special stage of life, was also examined with the IPAQ-SF. In a randomized controlled trial Amezcua-Prieto and co-authors determined the effect of walking and insomnia prevention in the third trimester, PA was measured by self-report using PAQ-SF and objectively with pedometers. Only the study protocol of the Walking Preg Project (WPP) has been published so far. The authors estimated that the promotion of walking in the second half of pregnancy by using a pedometer and pre-registering a goal to be achieved -'10,000–11,000 steps a day’- can prevent of insomnia in the third trimester and increase the quality of sleep and quality of life for pregnant women [20].

Subramaniam et al. studied the lifestyle factors, including poor dietary patterns, physical inactivity and their association with socio-demographic factors, clinical parameters, and health-related quality of life, in a Singapore in a cross-sectional study using the IPAQ-SF. The study was conducted among patients with multimorbidity in co-occurrence of two or more chronic health conditions (*N* = 932). Insufficiently active respondents were significantly more likely to be overweight/obese (OR: 1.5, 95% CI: 1.1–1.9, *p* = 0.01) than those who were sufficiently active. Based on a multiple linear regression model insufficient activity level was negatively associated with HRQoL (EQ-5D index score β = − 0.05, *p* < 0.001 and visual analogue scale β = − 4.4, *p* < 0.001) as compared to sufficient active levels in respondents with multimorbidity [21].

Due to its simplicity, the IPAQ-SF can be used in large population epidemiological studies with a high degree of reliability. In the Yazd Health Study (YaHS), population-based cohort study of Iranian adults (*N* = 9965), psychological and PA assessments were performed based on depression, anxiety and stress scale questionnaire (DASS 21) and the short form of the IPAQ. Finally, multiple logistic regression analysis was used to evaluate the relation between dietary intake and psychological disorders. However, in this study the main focus was not on PA, it was only included as a confounder [22].

Although IPAQ-SF is not a new questionnaire, several validation studies are being conducted to date [6, 14, 23–25]. It has been validated and is accessible on the IPAQ webpage in at least 18 languages so far, which allows a high level of comparability. This may be one of the reasons why the IPAQ-SF was selected in the framework of an important EU research and methodological project launched in January 2018, the “European Union Exercise and Sport Monitoring System”- EUPASMOS. The project, supported by the Erasmus + program, examines the exercise and sporting habits of the adult European population. The aim of the initiative to become a common, integrated system for monitoring exercise and in the participating EU Member States and beyond, throughout the European Union. The results of the current international PA and exercise habits questionnaires (Eurobarometer, European Public Health Survey, International Physical Activity Questionnaire – IPAQ and Global Physical Activity Questionnaire - GPAQ) are compared with the data from an objective measuring device (accelerometer) to find out which method is best suited for measuring exercise habits throughout Europe [26].

A systematic review by Lee et al. included twenty-three validation studies published in English, which studies validated the IPAQ-SF against an objective PA measuring device, doubly labelled water, or an objective fitness measure. Although the methods used in the studies found great variability, the results were largely similar. Correlations between the total PA measured by the IPAQ-SF and objective standards ranged from 0.09 to 0.39; none of the studies reached the minimum acceptable standard (0.50 for objective PA measuring devices, 0.40 for fitness measures). Correlations between the IPAQ-SF vigorous or moderate activity or walking and objective measures showed high variability (− 0.18 to 0.76), although several reached the minimal acceptable standard. A relationship was found between subjective measures and objective criterion in of six studies. Most studies overestimated PA with the self-reports by an average of 84% (36–173%); in contrast one of them underestimated it by 28%. Based on the above, the authors question the use of the IPAQ-SF as an indicator of relative or absolute PA [27].

However, there was a good correlation between the measurements regarding the reliability of test-retest (R = 0.788–0.981, *p* < 0.001) and the questionnaire showed a moderate agreement, with Cronbach Alpha value of 0.647. Based on the concurrent validity using RM42 accelerometers, we also found a significant, weak to moderate correlation (R = 0.111–0.338, *p* = 0.042; *p* < 0.001). Contrary to the opinion of Lee et al., we do recommend the IPAQ-SF as a compact, cost-effective, validated method for assessing physical activity. Particularly in the case of long combined questionnaires, the further extension of items, such as with the long form of IPAQ, would greatly reduce the response rate.

This study has a number of strengths and limitations that need to be considered when interpreting the results.

### Strengths

A similar study, which examined a relatively high sample using accelerometer, was never performed in Hungary. A further strength of the research may be, that it is an accelerometer-based comparative study that compares self-reports (subjective measures) with RM42 registered data (objective measures). The accuracy and reliability of the data is enhanced by the fact that an assisted data collection was carried out with trained researchers. An additional analysis based on a large heterogeneous sample will allow the results to be interpreted for different subpopulations.

### Limitations

The sample was not randomly selected and derived from two Hungarian regions (Budapest and South Transdanubia), which may distort the results. The RM42, as many accelerometers, have limited ability to detect activity while the wearer is cycling and it is not suitable for the measurement the of the measurement of swimming or doing other water based activities, which can be a distortion in estimating PA. Future validation studies should consider the use of doubly labelled water as a criterion that despite its high cost remains the recommended standard [11, 16].

## Conclusions

Summarizing our study results for the examined reliability and validity of the IPAQ-SF, the newly adapted subjective measurement tool is an acceptable method to measure PA. Self-reported PA and ST showed significant correlation with accelerometery based data, that allows researchers to support self-reported measurement tools to examine individuals’ PA levels when assessing correlations between PA and health or social outcomes. The IPAQ-SF had good test-retest reliability, but low to fair concurrent validity for vigorous, moderate activities and walking and sitting time, compared to the objective criterion measure among Hungarian adults.

## Data Availability

The dataset supporting the conclusions of this article is available from the corresponding author on reasonable request.

## Abbreviations

APE: Angle for postural estimation
BMI: Body mass index
DASS 21: Depression, Anxiety and Stress Scale
EHIS: European Health Interview Survey
EU: European Union
EUPASMOS: European Physical Activity and Sport Monitoring System
GPAQ: Global Physical Activity Questionnaire
IPAQ-LF: International Physical Activity Questionnaire-long form
IPAQ-SF: International Physical Activity Questionnaire-short form
PAQs: Physical activity questionnaires
SB: Sedentary behaviour
SD: Standard deviation
VPA: Vigorous physical activity
WHO: World Health Organization
WPP: Walking Preg Project
YaHS: Yazd Health Study
